# Identifying and Understanding Communities Using Twitter to Connect About Depression: Cross-Sectional Study

**DOI:** 10.2196/mental.9533

**Published:** 2018-11-05

**Authors:** Amber D DeJohn, Emily English Schulz, Amber L Pearson, E Megan Lachmar, Andrea K Wittenborn

**Affiliations:** 1 Department of Geography, Environment, and Spatial Sciences Michigan State University East Lansing, MI United States; 2 Department of Human Development and Family Studies Michigan State University East Lansing, MI United States; 3 Division of Psychiatry and Behavioral Medicine Michigan State University Grand Rapids, MI United States

**Keywords:** depression, Web-based, social connection, Twitter, tweet, online communities

## Abstract

**Background:**

Depression is the leading cause of diseases globally and is often characterized by a lack of social connection. With the rise of social media, it is seen that Twitter users are seeking Web-based connections for depression.

**Objective:**

This study aimed to identify communities where Twitter users tweeted using the hashtag #MyDepressionLooksLike to connect about depression. Once identified, we wanted to understand which community characteristics correlated to Twitter users turning to a Web-based community to connect about depression.

**Methods:**

Tweets were collected using NCapture software from May 25 to June 1, 2016 during the Mental Health Month (n=104) in the northeastern United States and Washington DC. After mapping tweets, we used a Poisson multilevel regression model to predict tweets per community (county) offset by the population and adjusted for percent female, percent population aged 15-44 years, percent white, percent below poverty, and percent single-person households. We then compared predicted versus observed counts and calculated tweeting index values (TIVs) to represent undertweeting and overtweeting. Last, we examined trends in community characteristics by TIV using Pearson correlation.

**Results:**

We found significant associations between tweet counts and area-level proportions of females, single-person households, and population aged 15-44 years. TIVs were lower than expected (TIV 1) in eastern, seaboard areas of the study region. There were communities tweeting as expected in the western, inland areas (TIV 2). Counties tweeting more than expected were generally scattered throughout the study region with a small cluster at the base of Maine. When examining community characteristics and overtweeting and undertweeting by county, we observed a clear upward gradient in several types of nonprofits and TIV values. However, we also observed U-shaped relationships for many community factors, suggesting that the same characteristics were correlated with both overtweeting and undertweeting.

**Conclusions:**

Our findings suggest that Web-based communities, rather than replacing physical connection, may complement or serve as proxies for offline social communities, as seen through the consistent correlations between higher levels of tweeting and abundant nonprofits. Future research could expand the spatiotemporal scope to confirm these findings.

## Introduction

Each day, 313 million global users share 500 million messages or tweets on the popular social networking site Twitter [1]. Within a 280 character limit, users can type a hashtag followed by a word or phrase to discuss a specific topic, link content about the same topic, or initiate dialogue on a topic. Twitter content is publicly available worldwide, providing valuable data for research, including the topic of mental health [2].

Each year in the United States, about 7% of adults suffer from depression, but only half seek professional help [3,4]. In many communities, mental disorders such as depression carry a social stigma, resulting in further isolation and lack of treatment [5]. Increasingly, individuals with depression are turning to Web-based communities for support [6]. Because Twitter is a popular networking platform, people often turn to it to connect about mental health issues such as depression [2]. In one study, researchers examined depressive symptoms based on the *Diagnostic and Statistical Manual of Mental Disorders* in tweets and found them characterized by low mood, fatigue, or loss of energy and problems with the social environment [7,8]. In another study examining the same hashtag as this study, #MyDepressionLooksLike, researchers found that the content of tweets included themes such as dysfunctional thinking, lifestyle challenges, social struggles, apathy and sadness, suicidal thoughts and behaviors, and seeking relief from depression [2].

Other research has focused on why users communicate about mental health through Web-based communities like Twitter. For example, the study #WhyWeTweetMH resulted in several themes for tweeting about mental health, including a sense of community, raising awareness, combating stigma, a space for expression, and coping and empowerment [9]. In a study by Park et al (2012) of about 20,000 tweets from approximately 15,000 users regarding depression, results revealed higher levels of tweets about users’ own depressive feelings or symptoms as opposed to comments on treatments or others’ symptoms [10]. A study from South Korea found that Twitter users who are not depressed view Twitter as a place to share information, whereas depressed Twitter users view it as a site meant for social awareness and emotional interaction [11]. Twitter users with depression “followed,” or subscribed to, other users who posted about emotional and everyday life activities; they found it uplifting to read other peoples’ positive tweets and reading about others’ depression caused them to feel less isolated [11]. However, other research reveals that use of social media can exacerbate mental health symptoms such as depression because of constant social comparison, bullying, suicide contagion, and other aspects [12-15]. While prior research has provided insight into individual-level predictors of seeking connection about depression on social media, less is known about community-level factors.

Possibly, lack of neighborhood amenities, such as parks and museums, may indicate fewer destinations for social interaction between residents. In this way, social bonds may be influenced by a neighborhood’s structural and functional characteristics [16]. Lack of amenities may also increase stress among residents who must exert extra effort to gain access to these resources [16]. Poorer neighborhoods may have lower levels of trust between neighbors and lower social support, in part because of blighted conditions in the built environment [16]. In fact, evidence suggests that signs of physical disorder (eg, vacant homes) can lead to feelings of hopelessness and diminish social relationships [17]. Weich et al found that depression was higher among people living in neighborhoods with an abundance of vacant lots and fewer amenities such as gardens and local shops [18].

Lack of neighborhood amenities and thus limited social interactions may be factors that influence use of Twitter for seeking connection about depression. To explore community characteristics that may lead to higher numbers of Twitter users seeking Web-based support, this study employed spatial data to understand how built and demographic features of communities relate to use of Twitter for depression support. Our findings may inform community efforts to increase social interaction and provide support for residents’ mental health. This study provides unique information that has not yet been analyzed by examining geographical location and community amenities in the context of tweets about depression.

## Methods

### Ethical Approval

Our research was determined to be Nonhuman Subjects research by the Michigan State University Institutional Review Board. All researchers involved received ethical training by the same institutional review board.

### Twitter Data

Tweets were downloaded through Twitter’s public streaming data [19]. Using NCapture, tweets were identified by the hashtag #MyDepressionLooksLike (QSR International, Burlington, MA, USA). This hashtag encouraged Twitter users with depression to share their experiences and connect with one another, making it useful in answering our research question.

Twitter restricted data capture to a random sample of 10% of total tweets from public content, and we compiled it into a database [2]. The temporal window for tweet-capture lasted from May 25 to June 1, 2016 (one week of the Mental Health Month), and tweets were pulled at 10:00 am Eastern Standard Time each weekday. Tweets meeting the following criteria were included: (1) each tweet was an original, not a retweet; (2) only one tweet per user; and (3) tweets with geolocations located within a designated 12-state northeastern study area. Thus, 104 tweets were included for analysis at the county level.

### County Demographic Data

To predict expected tweets by county, we compiled demographic data commonly associated with depression diagnoses. We collected demographic data from the 2010 Census and the American Community Survey 2015 5-year estimates [20,21], including age, gender, race, household status, and percent living in poverty. Depression has been found to be the highest among women, unmarried people, low-income persons, ethnic minorities, and people aged 15-44 years [18,22-28]. Thus, we compiled county percentages of single-person households, females, those aged 15–44 years, white population, and those living below the poverty line (see Table 1 for data sources).

### Community Characteristics Data

To understand the relationship between community characteristics and levels of tweeting, we compiled data for characteristics that could provide support, in-person treatment, opportunities for social interaction, or indicators of active community residents. These characteristics included parks or protected open spaces, places of worship, museums, active voter rates, mental health care providers, nonprofit organizations (organized by National Taxonomy of Exempt Entities code), K12 schools, and owner-occupied housing units. Likewise, we compiled data on characteristics that might hinder community support, including vacant housing units. Community characteristic data came from several sources (see Table 1). Rates were generated for most variables per 100,000 total population. K12 school rates were generated per 100,000 school-aged population. Percent of active voters was calculated as the percentage of the adult population actively voting in 2016.

Nonprofit organizations were sorted into groups by purpose using Stata v11.1 (StataCorp, College Station, TX, USA): health, human services, public and societal benefit, religion, and education. Counts of nonprofit organizations by group, places of worship, mental health care providers, and vacant and owner-occupied housing units were summed by county. A rate per 100,000 population was then calculated. The spatial locations of museums, K12 schools, parks, and county boundaries were mapped using ArcGIS v10.5 (Esri, Redlands, CA, USA) [29]. Counts per county were summed, and then a rate per 100,000 total population was calculated. Park areas (in miles^2^) were also calculated in ArcGIS, as a percentage of the county’s total land area.

Active voter totals were all from the fourth quarter of 2016. We aggregated active voter counts by county and then divided these counts by the active voter population. We then calculated the percent of active voters. For states that do not report active voter totals, we used that state’s voter turnout rate for the 2016 presidential election.

### Calculating a Tweeting Index Value

We assumed that, in theory, community levels of support seeking through Twitter should be predicted by demographic characteristics associated with depression and poor mental health [23,25-28]. Thus, we fitted a regression model to predict county-level tweet counts using those variables. Specifically, our multilevel Poisson regression model with robust SEs included independent predictors commonly associated with depression diagnoses including percent female, percent population aged 15-44 years, percent white, percent single-person households, and percent below poverty line, offset by the county population. The model also accounted for the clustering of counties within states.

Our focal interest was on understanding areas that have higher or lower tweeting than expected. So, after fitting the model, we calculated the difference between observed and expected counts and categorized these residuals into tweeting index value (TIV) tertiles, whereby 1=undertweeting, mean −0.37 (SD 0.53); 2=tweeting as expected, mean −0.02 (SD 0.01); and 3=overtweeting, mean 1.36 (SD 1.55). These TIVs were then used to examine community characteristic trends.

**Table 1:** Demographic and community characteristic data sources.

Variable	Data source^a^	Year
Percent aged 15-44 years	US Census Bureau	2010
Percent female	US Census Bureau	2010
Percent white	US Census Bureau	2010
Percent single-person household	US Census Bureau	2010
Percent below the poverty line	US Census Bureau (American Community Survey 5-year estimates)	2015
Owner-occupied housing unit rate	US Census Bureau (American Community Survey 5-year estimates)	2015
Vacant housing unit rate	US Census Bureau (American Community Survey 5-year estimates)	2015
Places of worship rate	Association of Religion Data Archives	2010
Mental health care providers rate	County Health Rankings & Roadmaps	2015
Museum rate	Institute of Museum and Library Services	2017
K12 schools per 100,000 children	United States Geological Survey	2016
Percent active voter	State Board of Electors	2016
Percent area occupied by park	State Geographic Information Systems Data Portals	Various
Nonprofit organizations rates	Urban Institute National Center for Charitable Statistics Data Archives	2005
School-aged population	US Census Bureau	2010
County boundaries	United States Geological Survey	2016

^a^Tweet counts were collected by the research team and are not included here. Nonprofits were broken down into National Taxonomy of Exempt Entities classes for analysis, but these classes are not shown here. Rates per 100,000 total population unless otherwise noted.

During analysis, we decided to test whether our results might be driven by inclusion of one county—Washington, DC—because this county appeared to be an outlier for several reasons. For example, while its museums and other community amenities are very high, the population tends to be younger, and tweet counts were the highest. For this reason, we conducted a sensitivity analysis whereby the above model was also fitted without DC included. However, our incident rate ratios (IRRs) changed <1% for all independent variables (see Multimedia Appendix 1); therefore, these results are not included here. All subsequent analyses included all 245 counties. All analyses were conducted using Stata v11 (StataCorp, College Station, TX, USA).

### Evaluation of Community Characteristics by Tweeting Index Values

The suite of community characteristics outlined previously was selected due to each factor’s potential role in promoting or hindering support, in-person treatment, opportunities for social interaction, or indicators of active community residents. We calculated means of each characteristic by TIV. We then calculated a ratio of TIV5:1 and a Pearson correlation coefficient (*r*) and *P* value.

## Results

**Figure 1:**
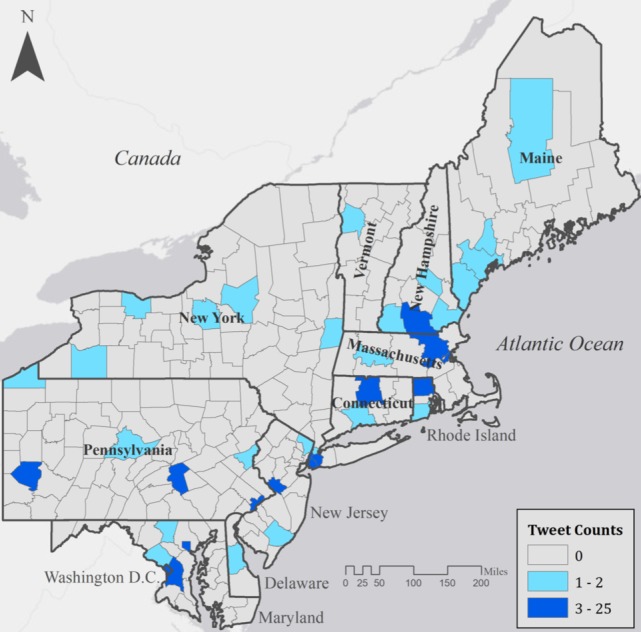
#MyDepressionLooksLike tweets by county for 11 states and the District of Columbia (2016).

Mapping captured #MyDepressionLooksLike tweets revealed that most counties in the study region were not using the hashtag during the observation period (Figure 1). Most tweets were in urban environments (eg, New York City and Washington, DC). Many counties near the eastern seaboard had higher numbers of tweets than counties farther inland. Delaware and Vermont each had only one county with a captured tweet.

Maryland, New York, and Pennsylvania had the highest tweet counts in our study region. However, their statewide means showed they were similar to other states. Washington, DC was an outlier in descriptive statistics of counties by state (Multimedia Appendix 2), having higher percentages of single-person households, people living below the poverty line, females, and population aged 15-44 years. New Hampshire had the lowest average percentage of single-person households (mean 15), while Connecticut had the lowest average percentage living below the poverty line (mean 9). Average county percentages of female population were similar across all states. Maine had the lowest average percentage population aged 15-44 years (mean 35). Washington, DC had the lowest percentage of white population (241,892/601,723, 40%), and Maine and Vermont had the highest average percentages of white population (mean 96).

**Figure 2:**
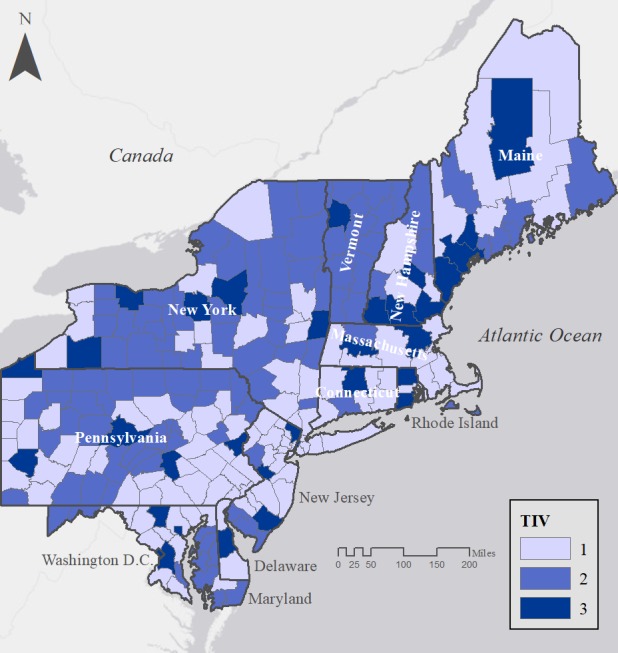
Tweeting index values by county for 11 states and the District of Columbia. TIV: Tweeting index values.

We found a positive, statistically significant correlation between several independent variables and tweet count (Table 2). These variables included percent population aged 15-44 years (IRR=1.11, *P*=.02), female (IRR=1.70, *P*<.001), and single-person households (IRR=0.90, *P*=.03). Percentages white and below poverty level were not statistically significant (Table 2). Regression model residuals were then used to create TIVs, which showed lower than expected tweets (TIV 1) in eastern seaboard areas and expected values (TIV 2) in western inland areas (Figure 2). Communities tweeting more than expected were generally scattered across our study region, with the exception of a cluster in the northern seaboard area.

Seeking to understand the relationship between the built environment and the levels of tweeting to connect about depression, we assembled a suite of community characteristics and related them to TIVs. We observed a *U-* shaped relationship between our TIVs and many of our community factors (see Table 3): rates of K12 schools, vacant housing, museums, places of worship, vacant housing rates, and health nonprofits. Almost all factors exhibiting *U* patterns also exhibited statistically significant correlations with TIV. In other words, areas with both undertweeting and overtweeting correlated with lower rates of these community characteristics. One exception was percent active voters, whereby lower values for TIV (undertweeting) correlated with higher rates of active voters. We also observed an upward gradient between TIV and rates of many types of nonprofits including all, human services, public benefit, religion, and education. Specifically, lower rates of nonprofits correlated with undertweeting and higher rates correlated with overtweeting.

**Table 2:** Regression results used to calculate tweeting index values (n=245) for counties in the study region.

Independent variables	Incident rate ratio	*P* value	95% CI
Percent aged 15-44 years	1.11	.02	1.02-1.21
Percent female	1.70	<.001	1.25-2.29
Percent white population	0.99	.37	0.96-1.02
Percent single-person household	0.90	.03	0.82-0.99
Percent below poverty level	1.05	.31	0.95-1.16

**Table 3:** Community characteristics by tweeting index values.

Community characteristics	TIV^a^1^b^	TIV2^b^	TIV3^c^	TIV3:TIV1	*r*	*P* value
K12 schools per 100,000 children, mean	199	289	212	1.07	0.19	.004
Museums rate, mean^d^	17	33	20	1.18	0.20	.002
Percent area occupied by park, mean	9	9	10	1.11	0.04	.49
Places of worship, mean^d^	104	177	93	0.89	0.16	.01
Vacant housing rate, mean^d,e^	6	17	7	1.17	0.16	.01
Owner-occupied housing rate, mean^d,e^	26	29	25	0.96	0.08	.21
Percent active voter, mean	76	72	74	0.97	-0.15	.02
Mental health care providers rate, mean^d^	209	157	293	1.40	0.09	.18
Nonprofits (all) rate, mean^d^	412	527	579	1.41	0.23	<.001
Nonprofits (health) rate, mean^d^	70	91	84	1.20	0.15	.017
Nonprofits (human services) rate, mean^d^	147	184	195	1.33	0.23	<.001
Nonprofits (public or societal benefit) rate, mean^d^	41	50	80	1.95	0.24	<.001
Nonprofits (religious) rate, mean^d^	12	14	17	1.42	0.13	.04
Nonprofits (education) rate, mean^d^	76	85	97	1.28	0.13	.04

^a^TIV: tweeting index values.

^b^Undertweeting.

^c^Overtweeting.

^d^Rate per 100,000 people.

^e^Values in thousands.

## Discussion

### Principal Findings

Most prior research on use of Twitter to connect about depression focused on interpersonal variables. This research focusing on community factors uniquely contributes to the literature. In this study, three positive, statistically significant associations were found to predict the count of tweets from people connecting about depression, including percent female, percent population aged 15-44 years, and percent single-person households. These findings correspond with other studies showing higher rates of depression among women and single-person households [18]. The percent female correlation further suggests that women experiencing depression are turning to Twitter at higher rates than men although previous research has shown no difference in general Twitter use between men and women [30].

While we found several significant associations, some anticipated predictors of tweet counts were not confirmed. Percent below poverty level and percent white were not significantly associated with tweet counts. Our insignificant findings for area-level poverty contrast with some published data showing an association between poverty and depression [31]. On the other hand, our findings echo those from Cutrona et al, whereby other neighborhood factors, such as social disorder, were more influential than poverty [16]. Prior results on depression and race have been inconsistent, with some studies indicating that the built environment may be more relevant to depression than race composition [32]. Other studies indicate that perceived discrimination, social support, and coping may play a role in the relationship between race and depression [33].

In examining community characteristics related to overtweeting or undertweeting, rates of K12 schools, museums, places of worship, vacant housing rates, and health nonprofits exhibited a *U-* shaped gradient across TIVs. Although Kaplan and Yen claim it difficult to illustrate a relationship between depression and lack of amenities, fewer amenities have been associated with higher depression rates within a neighborhood in prior research [34]. Our study reflected these difficulties because several community characteristics had a *U-* shaped relationship with TIVs. However, the rates of several types of nonprofits showed a clear, upward trend with TIVs, similar to other studies [17]. Lack of neighborhood amenities, such as community-building nonprofits, may not only be associated with higher rates of depression, but may also lead to lower levels of connecting about depression in Web-based communities. This suggests that Web-based communities, rather than replacing physical connection, act as complements and proxies for nonvirtual social communities. In other words, if communities have abundant nonprofit organizations serving the public, thereby improving community participation, residents experiencing depression may be more likely to connect about depression on the Web [35]. Conversely, if a community is lacking these amenities, the isolation that residents feel may transfer to their Web-based presence [16].

This paper expanded the literature about depression in relation to Twitter by exploring and providing information about community characteristics that correlate with people turning to a Web-based community to connect about depression. Before this study, literature about depression relating to Twitter mainly consisted of how to interpret depression based on tweets and why people tweet about their mental health issues, such as depression. This included findings underlying the positive impact of Web-based platforms such as Twitter in discussing mental health issues, but also pointed to social media use and adverse consequences, including increased depressive symptoms. While often overlooked, people’s environment strongly impacts the state of their mental health [36,37]. This study provided insight into aspects of a person’s environment, for instance, living in an area with many nonprofits, which may have encouraged Twitter use to bolster social connections.

### Limitations

Our study has several limitations. First, future research could expand the study area beyond the northeastern United States. As seen from Park et al’s 2013 study in South Korea, people from all over the world use Twitter to seek connections, so understanding their physical communities may also lend insight into the research question. Future studies could also expand the time frame from which tweets were gathered. Increasing the time frame for capturing data would allow this paper to have greater population validity and allow for inferences about how neighborhood changes relate to depression. Because of our limited spatiotemporal scope and use of NCapture, our sample size was smaller than originally anticipated, thus presenting generalizability concerns. Additionally, we used the geotagged location to characterize the community of Twitter users; this partially limited our sample and may have introduced error. Geotags may not be representative of the Twitter user’s community; rather, they could indicate places users were traveling to temporarily. Additionally, we used counties as a proxy for “community” since existing community-level data is often reported at the county level. In the future, we suggest a smaller geographic scale such as a city or town because these units are usually more representative of a person’s community.

### Conclusion

Our study is one of the first to explore built and social environmental contributions to the use of Twitter to connect about depression. Communities that overtweet and undertweet were more likely to have lower rates of K12 schools, museums, places of worship, vacant housing rates, and health nonprofits. These communities were also likely to have higher rates of active voters. Especially evident in our study is that communities with higher rates of nonprofits exhibited higher than expected levels of tweeting—suggesting that lack of community investment may influence Web-based connection seeking. Urban planning efforts may usefully promote amenities to bolster social interactions and lessen isolation, thus ultimately offering opportunities for social support for depression.

## Appendix

Multimedia Appendix 1 Tables used for sensitivity analysis.

Multimedia Appendix 2 Descriptive statistics for counties in study region by state.

## Abbreviations

IRR: incident rate ratio
TIV: tweeting index value
