# Supplementary material for: Identifying and Understanding Communities Using Twitter to Connect About Depression: Cross-Sectional Study
Source: JMIR Ment Health. 2018 Nov 5;5(4):e61. doi: 10.2196/mental.9533 (PMC6246977; doi:10.2196/mental.9533)
Supplement: Multimedia Appendix 1 [file mental_v5i4e61_app1.pdf]

Supplementary Materials for:

Identifying and Understanding Communities Using Twitter to Connect About Depression: Cross-Sectional Study

Amber D. DeJohn, Emily E. Schulz, Amber L. Pearson, E. Megan Lachmar, Andrea K. Wittenborn

This file contains additional tables used for sensitivity analysis, which excluded Washington, D.C.

Table S1. Sensitivity analysis - regression results excluding Washington, D.C.

Dependent variable: Tweet count<br>Independent variables:	IRR	*p* value	95% C.I.
**% aged 15-44**	1.11	.02	1.02, 1.21
**% female**	1.70	<.001	1.25, 2.31
**% white population**	0.99	.34	0.96, 1.02
**% single-person household**	0.90	.03	0.82, 0.99
**% below poverty level**	1.05	.30	0.95, 1.16

Table S2. Sensitivity Analysis - Community characteristics by TIVs excluding Washington, D.C.

Community Characteristics	Under-tweeting		Over-tweeting			
	TIV1	TIV2	TIV3	T3:T1	*r*	*P* value
**K12 schools per 100,000 children, mean**	200	290	212	1.06	0.19	.003
**Museums rate, mean[a]**	17	33	20	1.18	0.20	.002
**% Area occupied by park, mean**	8	9	10	1.25	0.05	.46
**Places of worship, mean[a]**	105	177	93	0.89	0.15	.017
**Vacant housing rate, mean[a,b]**	6	17	8	1.33	0.16	.01
**Owner-occupied housing rate, mean[a,b]**	26	29	25	0.96	0.08	.20
**% active voter, mean**	76	72	74	0.97	-0.14	.027
**Mental health care providers rate, mean[a]**	209	157	293	1.40	0.09	.17
**Non-Profits (all) rate, mean[a]**	412	528	579	1.41	0.23	<.001
**Non-Profits (health) rate, mean[a]**	70	91	84	1.20	0.15	.016
**Non-Profits (human services) rate, mean[a]**	147	184	195	1.33	0.23	<.001
**Non-Profits (public/societal benefit) rate, mean[a]**	41	50	80	1.95	0.24	<.001
**Non-Profits (religious) rate, mean[a]**	12	14	17	1.42	0.12	.057
**Non-Profits (education) rate, mean[a]**	76	85	97	1.28	0.13	.039

[a]Rate per 100,000 people

[b]Values in thousands
