# Supplementary material for: Identifying and Understanding Communities Using Twitter to Connect About Depression: Cross-Sectional Study
Source: JMIR Ment Health. 2018 Nov 5;5(4):e61. doi: 10.2196/mental.9533 (PMC6246977; doi:10.2196/mental.9533)
Supplement: Multimedia Appendix 2 [file mental_v5i4e61_app2.pdf]

Multimedia Appendix 2. Descriptive statistics for counties in study region, by state.

Descriptive statistics, mean (sd)	States in study region											
	CT	DE	DC[a]	ME	MD	MA	NH	NJ	NY	PA	RI	VT
**Tweet count**	0 (1)	0 (1)	8	0 (0)	1 (1)	1 (2)	1 (1)	0 (1)	1 (4)	0 (1)	1 (1)	0 (0)
**% single-person household**	18 (4)	21 (3)	24	16 (2)	20 (5)	18 (4)	15 (1)	20 (6)	18 (5)	16 (4)	17 (4)	16 (1)
**% below poverty level**	9 (2)	13 (1)	17	15 (3)	11 (5)	12 (4)	10 (2)	11 (5)	14 (4)	13 (3)	11 (4)	12 (2)
**% female**	51 (1)	52 (0)	53	51 (0)	51 (1)	52 (1)	51 (1)	51 (0)	50 (1)	50 (2)	52 (0)	50 (1)
**% by population aged 15-44**	39 (3)	38 (5)	52	35 (3)	39 (4)	39 (6)	37 (4)	39 (4)	39 (4)	37 (4)	38 (3)	36 (4)
**% white population**	83 (8)	71 (7)	40	96 (2)	72 (19)	84 (10)	95 (2)	73 (14)	86 (14)	92 (9)	89 (9)	96 (1)

[a]DC is a single observation, therefore mean and sd were not calculated.
